# Impact of staining on cell acoustic properties

**DOI:** 10.1016/j.bpj.2025.11.007

**Published:** 2025-11-10

**Authors:** Qing Wang, Taru Verma, Alexander Edthofer, Thierry Baasch, Thomas Laurell, Karina K. Sand, Andreas Lenshof, Wei Qiu

**Affiliations:** 1 Department of Biomedical Engineering, Lund University, Ole Römers väg 3A, 223 63 Lund, Sweden; 2 Section for GeoGenetics, Globe Institute, University of Copenhagen, Øster Voldgade 7, 1350 Copenhagen, Denmark

## Abstract

Cell staining techniques are essential for cell visualization and bioanalysis, providing valuable insights into cellular structure, function, and behavior. Despite their significance, the impact of staining processes, such as dye penetration, adsorption, or hydrogen bonding, on the physical properties of cells remains largely unexplored. Acoustic methods have proven effective in noninvasively probing various cell properties. In this study, we examine how different staining methods affect the acoustic properties of live DU145, MCF-7, Jurkat, and BV2 cell lines by comparing their acoustic impedance with and without staining. Our results show that calcein staining reduces the acoustic impedance of MCF-7 cells by 2.7%, whereas no noticeable changes are observed in other cases. This reduction, unique to MCF-7 cells, is most consistent with a modest increase in cell compressibility, potentially reflecting subtle alterations in membrane or cytoplasmic mechanics during dye and low-percent DMSO exposure.

## Significance

Cell staining is widely used to visualize cellular components, yet its impact on cell properties remains unclear. We systematically assess how different staining protocols affect the acoustic properties of live DU145, MCF-7, Jurkat, and BV2 cells. Membrane-binding stains (EpCAM, E-cadherin) and the cell-permeant nuclear dye Hoechst produce no detectable changes in acoustic impedance. In contrast, calcein staining cause a small but significant acoustic impedance reduction uniquely in MCF-7 cells, consistent with a modest increase in cell compressibility, i.e., mechanical softening at acoustic timescales, potentially linked to dye or low-percent DMSO exposure. These findings underscore the importance of considering staining-induced biophysical changes when interpreting experimental results, particularly in mechanobiology where even minor shifts in cellular mechanics can influence function and state.

## Introduction

Cell staining techniques are essential tools for enhancing contrast between cells and their surrounding medium. Various staining methods, including fluorescent labeling (1,2), immunohistochemistry (3), and dye staining (4), have been widely applied in biomedicine (5), pathology (6), and clinical diagnostics (7,8). Beyond simple visualization, these techniques enable real-time tracking of dynamic cellular processes such as division (9) and migration (10) while also assessing key indicators of cell status, including viability (11) and metabolic activity (12). Despite their proven utility in cell analysis, it remains largely unknown whether the staining process inadvertently affects cellular properties.

Staining reagents interact with cellular components (e.g., membrane, cytoplasm, and nucleus) through chemical reactions, hydrogen bonding, dye adsorption, and membrane penetration (13,14). For instance, Hoechst binds directly to DNA (15), antibodies cross-link to proteins on the cell membrane (16), and membrane-permeable calcein AM reacts with intracellular esterase in the cytoplasm (17). These interactions may induce subtle yet significant changes in nuclear stiffness, cytoskeletal organization, and metabolic activities, potentially skewing experimental outcomes and data interpretation. Notably, only a few studies have reported cytotoxic effects caused by calcein AM. Even at low concentration, calcein AM has demonstrated cytotoxic activity against human tumor cell lines (18). One study observed apoptotic responses resembling those induced by cytotoxic drugs, based on morphological changes occurring within hours of calcein AM exposure (19). These findings suggest that staining may alter cellular properties, yet systematic investigations quantifying these effects across different cell types and staining methods remain limited.

Cell physical properties, such as density, stiffness, deformability, and viscoelasticity, serve as critical biophysical markers for cell state, differentiation, and disease (20,21,22). These properties are highly sensitive to biochemical and physical perturbations, making them potential indicators of staining-induced alterations. Various methods have been developed to measure these properties, including atomic force microscopy (23,24), micropipette aspiration (25,26), optical stretcher (27,28), and magnetic tweezers (29,30), which can provide quantitative measurements. Among the available measurement techniques, acoustic-based approaches have gained increasing attention for probing physical properties of cells due to their noninvasive nature. Studies have demonstrated the use of these methods for measuring the compressibility of cell populations (31,32,33,34), as well as assessing stiffness (35), deformability (36), and effective acoustic impedance (37,38) at the single-cell level.

In this work, we investigate the effects of four different staining methods (i.e., calcein, EpCAM, E-cadherin, and Hoechst) on the physical properties of live DU145, MCF-7, Jurkat, and BV2 cell lines primarily by measuring their acoustic impedance. By comparing the acoustic impedance of stained and unstained cells, we aim to determine the extent to which staining alters cellular properties. Additionally, we examine how exposure to nonnutrient phosphate-buffered saline (PBS) solutions affects cell acoustic impedance. Through this systematic and quantitative evaluation, our study provides valuable insights into the biophysical impact of the staining process on cells.

### Theoretical background

Suspended cells exposed to a sound field experience both acoustic radiation force $F_{\text{rad}}$ and acoustic streaming. For a single spherical particle of radius *a* much smaller than the wavelength, in a half-wavelength standing-wave field along *y*-direction $e_{y}$ (in a coordinate system where y=0 is located at the pressure node), an analytical solution of $F_{\text{rad}}$ that accounts for medium viscosity has been derived (39,40). This solution is based on the earlier derivations for inviscid fluids (41) and is expressed as

(1a) $$F_{\text{rad}}\left(y\right)=-4\pi a^{3}k_{y}E_{\text{ac}}\Phi \left(\tilde{\kappa },\tilde{\rho },\tilde{\delta }\right)\sin\left(2k_{y}y\right)e_{y}\text{,}$$

(1b) $$\Phi \left(\tilde{\kappa },\tilde{\rho },\tilde{\delta }\right)=\frac{1}{3}f_{1}\left(\tilde{\kappa }\right)+\frac{1}{2}\text{Re}\left[f_{2}\left(\tilde{\rho },\tilde{\delta }\right)\right]\text{,}$$

where $k_{y}$, $E_{\text{ac}}$, and Φ represent the wavenumber, acoustic energy density, and acoustic contrast factor, respectively. The monopole and dipole scattering coefficients $f_{1}$ and $f_{2}$ are given by

(2a) $$f_{1}\left(\tilde{\kappa }\right)=1-\tilde{\kappa }\text{,}$$

(2b) $$f_{2}\left(\tilde{\rho },\tilde{\delta }\right)=\frac{2\left[1-\Gamma \left(\tilde{\delta }\right)\right]\left(\tilde{\rho }-1\right)}{2\tilde{\rho }+1-3\Gamma \left(\tilde{\delta }\right)}\text{.}$$

Here, the relative density and compressibility between the particle and the surrounding medium are given by $\tilde{\kappa }=\frac{\kappa_{p}}{\kappa_{m}}$, $\tilde{\rho }=\frac{\rho_{p}}{\rho_{m}}$, where $\kappa_{p}$ and $\kappa_{m}$ represent the compressibility of the particle and the medium, respectively, and $\rho_{p}$ and $\rho_{m}$ denote their densities. The effect of the medium viscosity $\eta_{m}$ is accounted for by the term $\Gamma \left(\tilde{\delta }\right)=-\frac{3}{2}\left[1+i\left(1+\tilde{\delta }\right)\right]\tilde{\delta }$, where $\tilde{\delta }$ is the ratio of the thickness of the viscous boundary layer, $\delta =\sqrt{\frac{2\eta_{m}}{\rho_{m}\omega }}$, to *a*. For cells exposed to a sound field at megahertz frequencies, the contribution of $\Gamma \left(\tilde{\delta }\right)$ is significantly smaller than that of $\tilde{\rho }$ (41). Once the particle begins to migrate, $F_{\text{rad}}$ balances the Stokes drag force $F_{\text{drag}}$, which in a quiescent medium is given by

(3) $$F_{\text{drag}}=-6\pi \eta_{m}av\text{.}$$

Thus, the $F_{\text{rad}}$ induced particle migration velocity in *y*-direction $v_{y}^{P}$ can be obtained by balancing (1a), (1b) and 3, which is expressed as

(4) $$v_{y}^{p}=-\frac{2a^{2}k_{y}E_{\text{ac}}\Phi \left(\tilde{\kappa },\tilde{\rho },\tilde{\delta }\right)\sin\left(2k_{y}y\right)e_{y}}{3\eta_{m}}\text{.}$$

Acoustic energy losses in the viscous boundary layers give rise to acoustic streaming, which also affects particle motion. In the ideal case, boundary-driven acoustic streaming is a vortical flow in the cross-sectional *y*-*z* plane. The velocity components in the *y*- and *z*-directions were analytically derived by Lord Rayleigh for a homogeneous fluid between two infinite parallel plates as (42)

(5a) $$\left\langle v_{2y}\right\rangle =\frac{3}{2}\frac{E_{\text{ac}}}{c_{m}\rho_{m}}\sin\left(2k_{y}y\right)\left[3\frac{{\left(2z\right)}^{2}}{H^{2}}-1\right]\frac{1}{2}\text{,}$$

(5b) $$\left\langle v_{2z}\right\rangle =\frac{3}{2}\frac{E_{\text{ac}}}{c_{m}\rho_{m}}\cos\left(2k_{y}y\right)\left[\frac{{\left(2z\right)}^{3}}{H^{3}}-\frac{2z}{H}\right]\frac{k_{y}H}{2}\text{,}$$

where *H* is the gap between the two plates with ceiling and bottom at $z=\pm \frac{H}{2}$.

## Materials and methods

### Cell culture and staining

In this study, we investigated the MCF-7 breast cancer cell line, the DU145 prostate cancer cell line, the Jurkat (T lymphocytes) cell line, and BV2 immortalized murine cell line. MCF-7 and DU145 cells are of epithelial origin, whereas Jurkat cells originate from connective tissue and BV2 cells from nervous tissue. Both Jurkat and DU145 cells were cultured in the recommended RPMI-1640 medium (Sigma-Aldrich, St. Louis, MO) with 10% fetal bovine serum (FBS, Sigma-Aldrich, non-US origin), which was supplemented with 1% penicillin-streptomycin-amphotericin B suspension (Sigma-Aldrich). MCF-7 cells were maintained in the HyClone Dulbecco's Modified Eagle Medium (DMEM) with 10% FBS, which was also supplemented with 1% penicillin-streptomycin-amphotericin B suspension. BV2 cells were cultured in DMEM (Sigma-Aldrich, St. Louis, MO) with 10% FBS and 1% penicillin-streptomycin-amphotericin B suspension. Cells were cultured in T75 cell culture flasks and incubated at 37°C in an incubator with a 5% CO_2_ atmosphere. They were passaged every 3–4 days by splitting. Adherent cells were detached from the flask using a trypsin solution and resuspended in cell culture medium for each experiment.

To examine the impact of staining on cell physical properties, four fluorescent dyes were used: a cell-permeable tracer calcein AM (Fisher Scientific International, Pittsburgh, PA) for cytoplasm staining; anti-EpCAM FITC (Becton, Dickinson and Company, Franklin Lakes, NJ), which binds to the cell membranes; E-cadherin CD324 PE (Miltenyi Biotec Norden, Lund, Sweden), also for cell membrane binding; and Hoechst 33342 (TargetMol Chemicals, Boston, MA), which binds to DNA in the cell nucleus. To prepare the stained cells, they were first resuspended in PBS after centrifugation at 200 × *g* for 5 min. Calcein staining was performed by incubating the cells on ice in the dark for 20, 40, and 60 min in PBS containing 2 μM calcein AM. EpCAM staining was carried out by incubating the cells at room temperature in the dark for 25 min in PBS with 0.86 μg/mL EpCAM. The E-cadherin staining was achieved by incubating the cells in a refrigerator in the dark for 10 min in PBS with E-cadherin (PBS to E-cadherin volume ratio of 98:2). Hoechst staining was performed by incubating the cells at room temperature in dark for 10 min in PBS containing 10 μM Hoechst. After staining, the cells were washed by centrifugation and resuspended in PBS at a concentration of $1.4\times 10^{6}$ cells per mL. The sizes of both stained and unstained cells were measured using a Coulter counter (Beckman counter, Brea, CA), as summarized in Table S1 of the supporting material.

### Preparation of density-modified cell medium

OptiPrep (STEMCELL Technologies, Norway) containing 60% w/v iodixanol was used to adjust the acoustic impedance of the medium with minimal impact on viscosity. Iodixanol solutions with concentrations ranging from 4% to 31% were prepared by diluting 60% iodixanol with PBS. Both stained and unstained cells were directly mixed with the iodixanol solutions to create density-modified cell suspensions before each measurement. The density and speed of sound of each solution were measured at room temperature using a density and sound velocity meter (DSA 5000 M, Anton Paar, Graz, Austria), as shown in Fig. S1 of the supporting material.

### Experimental setup

**Figure 1:**
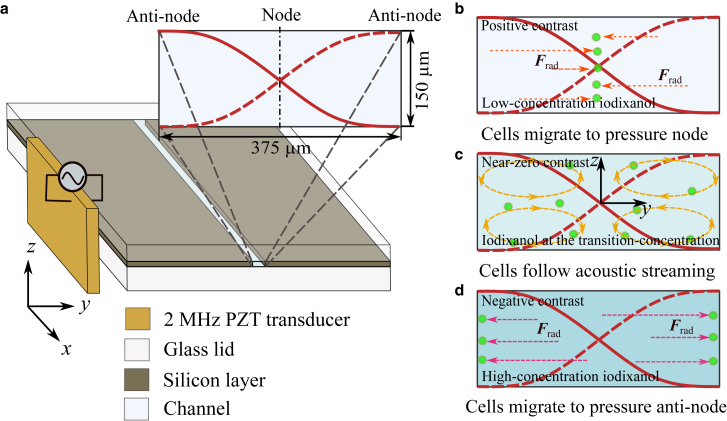
Schematic illustration of the measurement principle. (*a*) The chip used in this study consisted of a silicon layer sandwiched between two glass layers through anodic bonding. A straight channel with a rectangular cross section (375 × 150 $\mu m^{2}$) was etched through the silicon layer. A 1-mm-thick PZT transducer was attached to the chip sidewall to generate a half-wavelength standing-wave field along the channel width, *y*-direction, at approximately 2 MHz. When cells are suspended in homogenous iodixanol solutions, their migration behavior can be classified into three categories based on their acoustic contrast factors: (*b*) positive, (*c*) zero, and (*d*) negative acoustic contrast factors. For cells with positive or negative contrast factors, migration is dominated by the acoustic radiation force; hence, they move to either pressure node (channel center) or antinodes (channel sidewalls). In contrast, cells with a near-zero contrast factor primarily follow the acoustic streaming rolls, resulting in their circulation in the channel.

Standard glass-silicon-glass chips featuring a straight channel of 45×0.375×0.15 mm^3^ were fabricated by deep reactive-ion etching, as illustrated in Fig. 1 *a*. The channel was sealed by two anodically bonded glass lids, with thickness of 500 μm and 610 μm, respectively. A 1-mm-thick lead zirconate titanate (PZT) transducer was attached to the sidewall of the chip (43). It was driven by a function generator (AFG3022B, Tektronix, Beaverton, OR) to excite a half-wavelength standing-wave field along the channel width at approximately 2 MHz.

The chip was mounted on an aluminum holder, and cell migrations under an acoustic field were observed using an inverted fluorescence microscope (Eclipse Ti2, Nikon, Tokyo, Japan) equipped with a CMOS camera. Images of unstained cells and cells stained with E-cadherin and EpCAM were captured using phase-contrast imaging under white-light LED illumination, whereas images of calcein- and Hoechst-stained cells were acquired through fluorescence imaging under blue LED illumination.

### Experimental procedure

After being harvested from the cell culture medium, cells were resuspended in PBS. Since PBS may influence the physical properties of cells, a fair comparison was ensured by measuring the acoustic impedance of both stained and unstained cells simultaneously in two acoustofluidic devices. These measurements were conducted between 1 and 3 hours after suspension in PBS. Before each acoustic focusing experiment, the cell suspension was mixed with iodixanol solutions of varying concentrations. The cells were then introduced into the channel, and the flow was stopped using a two-port valve. Their motion was recorded once the sound field was turned on. After image acquisition, the cells exposed to the acoustic field were flushed out, and fresh cells were infused to the channel. This process was repeated 15 to 20 times to collect a sufficient number of cells in each iodixanol solution. The total measurement time for each iodixanol solution was kept below 10 min.

## Results and discussion

### Determination of cell acoustic impedance

**Figure 2:**
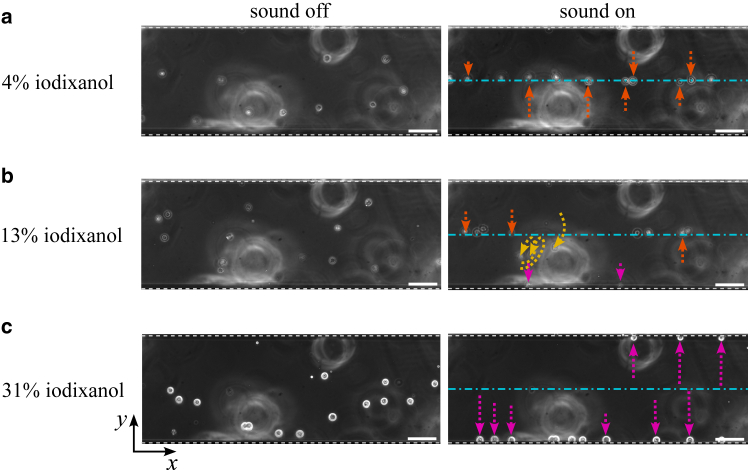
Phase-contrast images of unstained MCF-7 cells suspended in iodixanol solutions at concentrations of (*a*) 4%, (*b*) 13%, and (*c*) 31%. The arrows indicate the cells migrated toward the pressure node (*orange*), the antinodes (*magenta*), or followed the acoustic streaming (*yellow*) when exposed to a half-wavelength standing-wave field. The cyan dashed line represents the pressure node, whereas the white dashed lines mark the pressure antinodes located at the channel sidewalls. The scale bar represents 100 μm.

When cell motion is primarily governed by the acoustic radiation force $F_{\text{rad}}$, cells migrate either to the pressure node if they have a positive acoustic contrast factors Φ (Fig. 1 *b*) or to the antinode if Φ is negative (Fig. 1 *d*). When Φ is close to zero, the velocity induced by $F_{\text{rad}}$ becomes negligible, and cell motion is instead dominated by acoustic streaming, as illustrated in Fig. 1 *c*. At Φ=0, the effective acoustic impedance of the cell, $Z_{c}=\sqrt{\rho_{c}/\kappa_{c}}$ where $\rho_{c}$ and $\kappa_{c}$ are cell density and compressibility, is approximately equal to that of the surrounding medium $Z_{m}$ (37). It is worth noting that $Z_{c}$ is an intrinsic physical property of the cells, whereas Φ is a relative measure that depends on both the properties of the cells and those of the surrounding medium. For a group of cells of the same type, variations in cell motion in the same medium may still occur due to the heterogeneity in their physical properties. In this study, iodixanol solutions of varying concentrations were prepared, with higher concentrations corresponding to increased acoustic impedance. At low iodixanol concentrations, most cells exhibit a positive Φ and migrate toward the pressure node, as shown in Fig. 2 *a*. As the concentration increases, the impedance mismatch between the $Z_{c}$ and $Z_{m}$ decreases, causing Φ to transition from positive to negative. In the medium where this transition occurs, cells may migrate to node, antinodes, or follow the streaming (Fig. 2 *b*). At even higher concentrations, Φ becomes negative for most cells, leading to equilibrium positions at the antinodes (Fig. 2 *c*).

**Figure 3:**
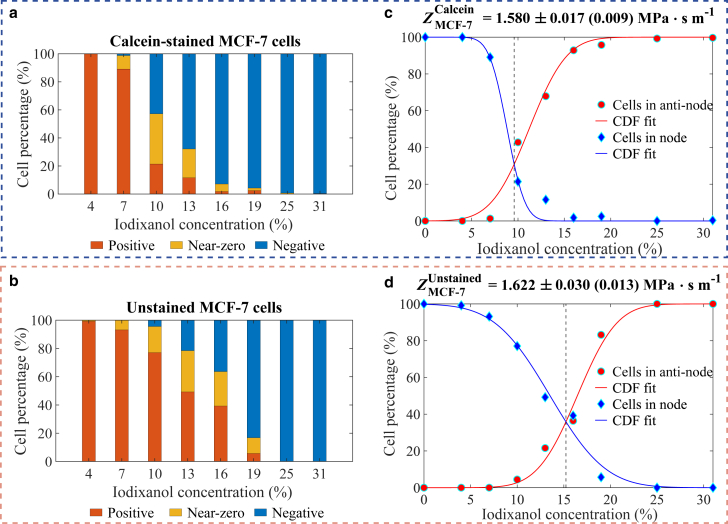
Determination of the acoustic impedance of calcein-stained and unstained MCF-7 cells. (*a*) and (*b*) present the percentages of cells exhibiting positive, zero, and negative contrast factors across different iodixanol concentrations. (*c*) and (*d*) display the cell percentages at the pressure node (*blue dots*) and antinodes (*red dots*), with data fitted to the cumulative distribution function of a normal distribution. The transition iodixanol concentration is identified at the intersection of the two fitting curves, where the proportion of cells with contrast factor near zero is maximized. The acoustic impedance was determined to be 1.580 ± 0.017 (0.009) MPa · s m^−1^ (mean ± standard deviation (fitting error of the mean)) for calcein-stained MCF-7 cells and 1.622 ± 0.030 (0.013) MPa · s m^−1^ for unstained MCF-7 cells.

To determine the effective acoustic impedance of cells, $Z_{c}$, the number of cells migrating to the pressure node, antinodes, or following the acoustic streaming was counted in each iodixanol solution. The percentage of each migration type relative to the total cell count was then calculated, as illustrated in Fig. 3 *a* and *b*. The percentages of cells migrating to the pressure node and antinodes were fitted to the cumulative distribution function (CDF) of a normal distribution, represented as y=1−CDF(μ,σ) and y=CDF(μ,σ), respectively, as shown in Fig. 3 *c* and *d*. The intersection point of these two fitted curves represents the transition iodixanol concentration, where the percentage of cells with Φ close to zero is the highest. Consequently, the mean $Z_{c}$ can be determined using the relationship $Z_{c}\approx Z_{\text{Idx}}^{\text{Trans}}$, where $Z_{\text{Idx}}^{\text{Trans}}$ is the acoustic impedance of iodixanol at the transition concentration (38). The results indicate that the transition iodixanol concentration for calcein-stained MCF-7 cells was 9.6%, whereas it was 15.2% for unstained MCF-7 cells, as shown in Fig. 3 *c* and *d*. This shift in transition iodixanol concentration reflects a reduction in acoustic impedance from 1.622 ± 0.030 (0.013) MPa · s m^−1^ to 1.580 ± 0.017 (0.009) MPa · s m^−1^ (mean ± standard deviation (fitting error of the mean)) after calcein staining.

### The effect of PBS solution on cell acoustic impedance

**Figure 4:**
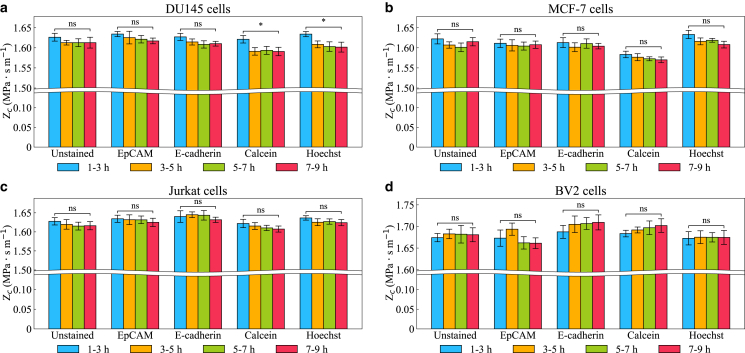
Measured acoustic impedance of stained and unstained (*a*) DU145, (*b*) MCF-7, (*c*) Jurkat, and (*d*) BV2 cells over 9 hours after exposure to PBS solution. The incubation time for calcein-stained cells was 20 min. Colors represent the acoustic impedance measured at different time points, whereas error bars indicate the fitting error of the mean obtained from fitting the data to the cumulative distribution function of a normal function. Brackets indicate endpoint contrasts (1–3 h to 7–9 h) tested with two-sided Wald z-tests on the two fitted estimates. Asterisks denote significance (^∗^*p* < 0.05; ns = not significant). *p*-values reflect within-experiment uncertainty of the fits.

Since cellular properties can change over time after leaving the nutrient-rich culture medium and entering the PBS solution, the acoustic impedance of cell populations was measured at different time points (1–9 h) after their transfer to PBS, as shown in Fig. 4. We observed small time-dependent decreases in acoustic impedance for DU145 cells using calcein and Hoechst staining after suspension in PBS (see Fig. 4 *a*). Mechanistically, cellular processes that follow nutrient deprivation, for example autophagy (44) or partial depolymerization of cytoskeletal filaments (45), could lower $Z_{c}$ by either reducing bulk density (loss or redistribution of macromolecular content) or increasing compressibility (greater volumetric compliance of the cell interior). In practice, however, our time-course measurements show that these effects are modest and not consistent across cell lines or stains. Only calcein- and Hoechst-stained DU145 cells showed nominally significant changes over the 1- to 9-h window, whereas other conditions did not. Given the small effect sizes and limited statistical robustness, we avoid strong mechanistic claims.

### Impact of different staining on cell acoustic properties

**Figure 5:**
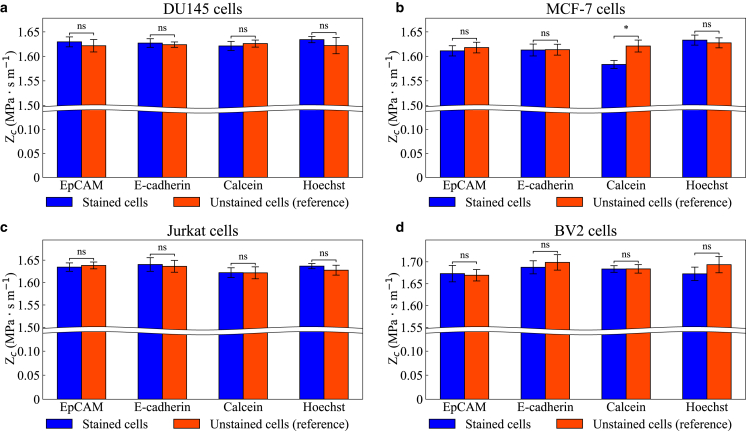
Measured acoustic impedance of (*a*) DU145, (*b*) MCF-7, (*c*) Jurkat, and (*d*) BV2 cells stained using four different protocols. The incubation time for calcein-stained cells was 20 min. Orange bars represent the acoustic impedance of unstained cells measured in parallel with the stained cells. All measurements were conducted between 1 and 3 hours after suspension in PBS solutions. Error bars indicate the fitting error of the mean obtained from fitting the data to the cumulative distribution function of a normal function. Brackets indicate contrasts (unstained and stained) tested with two-sided Wald z-tests on the two fitted estimates. Asterisks denote significance (^∗^*p* < 0.05; ns = not significant). The *p*-values reflect within-experiment uncertainty of the fits.

**Figure 6:**
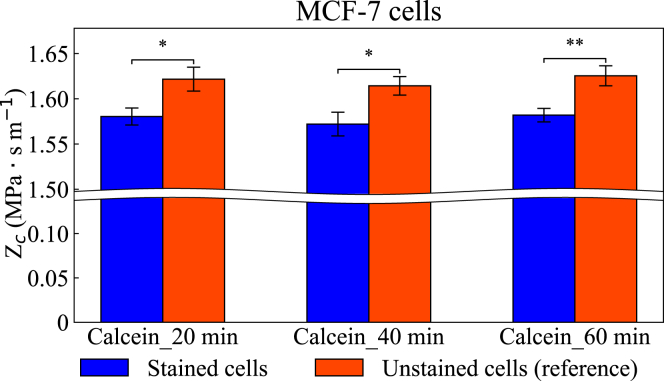
Measured acoustic impedance of calcein-stained MCF-7 cells at different incubation times. Orange bars represent the acoustic impedance of unstained cells measured in parallel with the stained cells. All measurements were conducted between 1 and 3 hours after suspension in PBS solutions. Error bars indicate the fitting error of the mean obtained from fitting the data to the cumulative distribution function of a normal function. Brackets indicate contrasts (unstained and stained) tested with two-sided Wald z-tests on the two fitted estimates. Asterisks denote significance (^∗^*p* < 0.05; ^∗∗^*p* < 0.01). The *p*-values reflect within-experiment uncertainty of the fits.

To account for the potential effect of PBS solution on the acoustic impedance of a cell and ensure a fair comparison, the acoustic impedance of stained and unstained cells was measured simultaneously (1–3 h after the exposure to PBS) by two operators using two identical acoustofluidic devices. The acoustic impedance of unstained cells served as a reference for comparison of stained cells, and the results are summarized in Fig. 5. Among the four cell types tested, staining with EpCAM, E-cadherin, or Hoechst did not cause any noticeable change in acoustic impedance. For EpCAM and E-cadherin, this is consistent with their binding only to membrane antigens without penetrating the cytoplasm. Hoechst, in contrast, is a cell-permeant nuclear dye, but at the concentrations used here, its binding to DNA did not measurably affect cell density or compressibility, resulting in no detectable change in acoustic impedance. Interestingly, calcein-stained MCF-7 cells exhibited a reduction in acoustic impedance of up to 2.7%, as shown in Fig. 5 *b*, whereas the same staining method did not produce a clear change in DU145, Jurkat, or BV2 cells. Despite the change in acoustic impedance, calcein staining did not affect cell size, as shown in Table S1. The effect of the incubation time for calcein-stained cells with respect to the acoustic impedance was also investigated (see Fig. 6), with incubation periods of 20, 40, and 60 min, based on the manufacturer’s recommendation for calcein AM reagent. The results indicate that varying incubation time did not significantly affect the acoustic impedance of the investigated cells.

Using $Z_{c}=\sqrt{\rho_{c}/\kappa_{c}}$ and a first-order expansion $\frac{\Delta Z_{c}}{Z_{c}}\approx \frac{1}{2}\left(\frac{\Delta \rho_{c}}{\rho_{c}}-\frac{\Delta \kappa_{c}}{\kappa_{c}}\right)$, the observed 2.7% decrease in acoustic impedance corresponds to a 5.4% increase in compressibility if density is approximately constant. A density-only explanation would require a 5.4% reduction in $\rho_{c}$, which is implausible given the negligible added solute mass fraction during staining ($<10^{-6}$). Since cell size remained unchanged, osmotic effects or major cytoplasmic loss are unlikely. We therefore interpret the shift primarily as a modest increase in compressibility while acknowledging that a minor density contribution cannot be excluded.

Calcein AM (19,46) is highly lipophilic, firstly partitions into the membrane during loading and is then hydrolyzed by intracellular esterases into fluorescent calcein, and is widely used as a viability and esterase activity probe (47). As esterases are not generally considered regulators of lipid metabolism, we avoid causal claims linking this process to lipid regulation. Instead, the selective response of MCF-7 cells may reflect lipid-phase perturbations during dye exposure and the presence of low-percent DMSO (0.1% v/v), routinely used to deliver calcein AM. DMSO at such concentrations is known to modulate membrane structure and fluidity (48,49). Moreover, breast cancer cells, including MCF-7, undergo lipid-metabolic reprogramming (50) and exhibit variable lipid-droplet content (51), which could render them more sensitive to transient lipid-phase perturbations than the other cell lines tested. Thus, differences in lipid physiology combined with short-term dye/DMSO exposure may plausibly contribute to the small increase in compressibility observed uniquely in calcein-stained MCF-7 cells.

## Conclusion

In this study, we examined how different staining methods affect the acoustic properties of live cells by comparing their acoustic impedance with and without staining. We found that EpCAM, E-cadherin, and Hoechst staining caused no measurable changes across the tested cell types, indicating minimal influence on cell density or compressibility. In contrast, calcein staining produced a small but significant reduction in acoustic impedance uniquely in MCF-7 cells, which is most consistent with a modest increase in compressibility of the cell interior. These results highlight that although most stains do not perturb cellular acoustic properties, certain dyes can induce subtle yet detectable biophysical changes that may influence downstream mechanobiological assays. Our measurements probe acoustic properties in the low-megahertz regime, providing insight into bulk compressibility at high frequencies. An interesting future direction would be to perform complementary measurements of quasistatic elastic moduli, for example using atomic force microscopy, to compare cell mechanics across two very different frequency regimes. Moreover, the potential link between high-frequency acoustic properties and functional cellular mechanics such as proliferation, differentiation, and mechanotransduction remains largely unexplored. Establishing this connection will require systematic studies but may provide important new insights into how cellular mechanics operate across timescales from acoustic to quasistatic.

## Acknowledgments

We thank Sara Florentzson at Lund University for her initial measurements of the acoustic mobility ratio of stained and unstained cells relative to 5-μm-diameter polystyrene particles. Q.W. was supported by the Foreign Postdoctoral Fellowship from 10.13039/100014437Wenner-Gren Foundations (grant no. UPD2022-0137). T.B. was supported by the grant for scientific research from the Crafoord Foundation (grant no. 20240891) and the Starting Grant from 10.13039/501100004359Swedish Research Council (grant no. 2022-04041). T.L. was supported by the Distinguished Professor Grant from 10.13039/501100004359Swedish Research Council (grant no. 2019-00795). W.Q. was supported by the Starting Grant from 10.13039/501100004359Swedish Research Council (grant no. 2021-05804) and the grant for scientific research from the Crafoord Foundation (grant no. 20241032).

## Author contributions

W.Q. conceived the idea. Q.W., A.L., and W.Q. designed the experiments. Q.W. and A.L. cultured and prepared the cells. Q.W., T. V., and W.Q. conducted the experiments. All authors analyzed and discussed the data. Q.W. and W.Q. wrote the manuscript with input from all authors.

## Declaration of interests

Thomas Laurell is the founder, chairman of the Board, and shareholder of AcouSort AB, a company that commercializes acoustofluidic technology.

## Supporting material

Document S1. Figure S1 and Table S1

Document S2. Article plus supporting material
