# Supplementary material for: Impact of staining on cell acoustic properties
Source: Biophys J. 2025 Nov 10;125(1):77–85. doi: 10.1016/j.bpj.2025.11.007 (PMC12821031; doi:10.1016/j.bpj.2025.11.007)
Supplement: Document S1. Figure S1 and Table S1 [file mmc1.pdf]

Biophysical Journal, Volume *125*

Supplemental information

Impact of staining on cell acoustic properties

Qing Wang, Taru Verma, Alexander Edthofer, Thierry Baasch, Thomas Laurell, Karina K. Sand, Andreas Lenshof, and Wei Qiu

Supplementary Material

Impact of Staining on Cell Acoustic Properties

Qing Wang [1], Taru Verma [2], Alexander Edthofer [1], Thierry Baasch [1], Thomas Laurell [1], Karina K. Sand [2], Andreas Lenshof [1], and Wei Qiu [1, *]

[1] Department of Biomedical Engineering, Lund University, Ole Römers väg 3A, 223 63 Lund, Sweden
[2] Section for GeoGenetics, Globe Institute, University of Copenhagen, Øster Voldgade 7, 1350 Copenhagen, Denmark
*Correspondence: wei.qiu@bme.lth.se

S1. Sizes of stained and unstained cells

The sizes of stained and unstained cells were measured using a Coulter counter. The data were fitted with a Gaussian function to determine the mean and size distribution. A summary of the mean values and size distributions for both stained and unstained cells is presented in Table S1.

Table S1 Measured sizes (mean value and size distribution) of stained and unstained cells.

Cell type	Staining reagent	Mean size (μm)	Distribution (μm)
DU145	Unstained	20.5	4.4
	Calcein AM	20.8	4.7
	Hoechst	19.5	2.6
	EpCAM	20.5	4.4
	E-cadherin	20.3	4.5
MCF-7	Unstained	21.7	6.3
	Calcein AM	21.5	5.0
	Hoechst	19.2	3.2
	EpCAM	20.5	7.1
	E-cadherin	20.7	7.6
Jurkat	Unstained	17.6	3.7
	Calcein AM	17.1	2.8
	Hoechst	17.1	2.1
	EpCAM	16.7	2.9
	E-cadherin	16.6	3.1
BV2	Unstained	15.0	1.7
	Calcein AM	14.7	1.6
	Hoechst	15.3	1.5
	EpCAM	14.6	1.7
	E-cadherin	15.0	1.4

S2. Acoustic properties of iodixanol solutions

The density (ρ_{m}) and speed of sound (c_{m}) of iodixanol solutions at varying concentrations were measured using a density and sound velocity meter (DSA 5000 M, Anton Paar GmbH, Graz, Austria) at 25 ℃. The corresponding acoustic impedance Z_{m} was calculated as $Z_{\mathrm{m}} = \rho_m c_{\mathrm{m}}$. Results showed that Z_{m} increased linearly with iodixanol concentration within the studied range, as illustrated in Figure S1. The Z_{m} at the transition concentration was determined using the linear relationship $y = 0.7318x + 1.5102$.

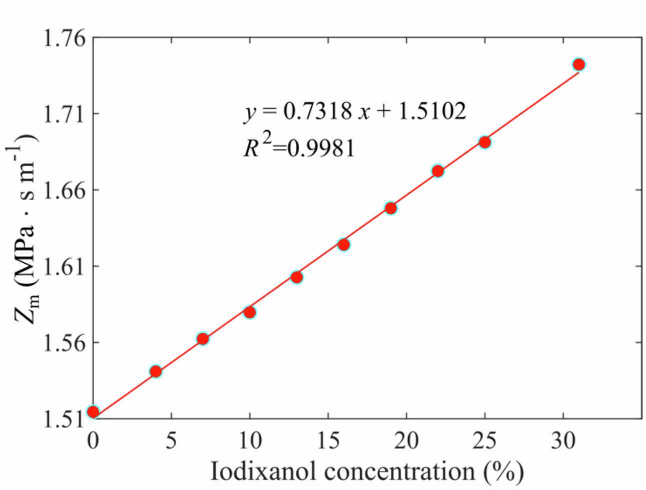

Figure S1. The dependance of medium acoustic impedance Z_{m} on iodixanol concentration. The data were fitted by a first-order polynomial function.
